# Supplementary material for: Metabolomics analysis of the yolk of Zhijin white goose during the embryogenesis based on LC-MS/MS
Source: PLoS One. 2024 Feb 9;19(2):e0297429. doi: 10.1371/journal.pone.0297429 (PMC10857567; doi:10.1371/journal.pone.0297429)
Supplement: Attachment — Submitted filename: Responses for reviewers.docx [file pone.0297429.s004.docx]

**List of Responses**

Dear editors and reviewers:

Thank you very much for your careful review and constructive suggestions with regard to our manuscript “Metabolomics analysis of the yolk of Zhijin white goose during the embryogenesis based on LC-MS/MS (PONE-D-23-38791R1)”. We have carefully evaluated the Editors/Reviewers’ critical comments and thoughtful suggestions, responded to these suggestions point-by-point, and revised the manuscript accordingly. The revised parts in the manuscript have been marked in red. We appreciate for Editors/Reviewers’ warm work earnestly, and hope that the corrections will meet with approval. Please feel free to contact us with any questions and we are looking forward to your consideration. The main corrections in the manuscript and the responses to the Editors/Reviewers’ comments are as follows.

1. On Page 2, Line 26, please correct E (7) and define the numbers.

**Response:** Thank you very much, we have corrected E (7) and defined the numbers.

2. In abstract, authors have written that yolk metabolites were approximately similar at E7 and E12, E23 and E28 but what about E18.

**Response:** According to the advice, we have made appropriate changes to this sentence and added a note about E18.

3. Abbreviations used first time in the text should be explanatory (Page 3, Line 56).

**Response:** According to the advice, we have explained the acronyms EPA and DHA.

4. The format of heading should be uniform throughout manuscript.

**Response:** Based on the PLOS ONE style template, we have changed the level 1 headings to 18pt font and bold type, and the level 2 headings to 16-point font and bold type throughout manuscript.

5. Please cite the references form where extraction procedure and other methodologies were adopted.

**Response:** Thank you very much, we have cited the corresponding references in the section on metabolite extraction and data processing.

We have tried our best to improve the manuscript and made some changes in the manuscript. We appreciate for Editors/Reviewers’ warm work earnestly, and hope that the correction will meet with approval. Once again, thank you very much for your comments and suggestions.

Yours sincerely
